# Efficacy of Lazolex® Gel in the Treatment of Herpes Simplex Mucocutaneous Infections and the Prevention of Recurrences: A Pilot Study

**DOI:** 10.1155/2022/4413679

**Published:** 2022-11-16

**Authors:** Tina A. Kituashvili, Vakhtang G. Kvirkvelia, George G. Galdava, Nino G. Archvadze

**Affiliations:** ^1^Dermato-venereology Department, Medical Faculty, Tbilisi State University, Tbilisi 0159, Georgia; ^2^S/R National Centre of Dermatology and Venereology, Tbilisi 0154, Georgia; ^3^Division of Applied Biosciences and Biotechnology, Biology Department, Tbilisi State University, Tbilisi 0147, Georgia

## Abstract

**Background:**

Previous in vitro and in vivo studies indicated that walnut extract has a therapeutic effect on herpes simplex infections. This study aimed to evaluate the efficacy and tolerance of Lazolex® Gel (Iveriapharma, Tbilisi, Georgia), an emollient gel to treat mucocutaneous lesions caused by herpes simplex virus.

**Methods:**

A single-center, single-arm, open-label, phase II clinical trial was conducted with 30 patients divided into two groups: 15 patients with herpes simplex virus type 1 (HSV-1) infections and 15 with herpes simplex virus type 2 (HSV-2). All received topical treatment with Lazolex® Gel four times a day for 10 days. The efficacy and tolerance of the treatment were evaluated on day 10 and day 20 of the study. Recurrence rates were also evaluated both prior to treatment with Lazolex® and over a 4-year follow-up period subsequent to treatment.

**Results:**

The median effective time to resolution of symptoms (itching, burning, and pain) was 1.97 days in the HSV-1 group and 3.11 days in the HSV-2 group. The median effective time for vesicles and erosion to disappear was 3.64 days in the HSV-1 group and 3.88 days for the HSV-2 group. Finally, the median effective time for inflammatory signs to disappear was 5.70 and 4.32 days, respectively. Following treatment with Lazolex® Gel, the frequency of outbreaks decreased from a median of 2.00 and 1.00 times per year in the HSV-1 and HSV-2 cohorts to 0.25 and 0.00 (*p*=0.001 and *p*=0.003), respectively.

**Conclusions:**

Topical treatment with Lazolex® Gel applied to lesions four times a day for 10 days was shown to be effective and safe in the treatment of herpes simplex mucocutaneous infections and dramatically reduced the rate of recurrence. Clinical trial was approved by Drug Agency of Ministry of Labour, Health and Social Affairs of Georgia, registration # DA Nº CT-000032, date of approval 01.10.2007.

## 1. Introduction

Herpes simplex virus (HSV) belongs to the *Herpesviridae* family and the *Alphaherpesvirinae* subfamily [1]. HSV mainly affects the skin and mucosal membranes [2]. There are two types of HSV: type 1 (HSV-1), which is transmitted primarily by oral-to-oral contact and commonly causes herpes labialis (also known as orofacial herpes, cold sores, or fever blisters), and type 2 (HSV-2), which is predominantly transmitted sexually (it is considered a Sexually Transmitted Infection) and constitutes the leading cause of genital lesions [2–5]. In exceptional cases, HSV-1 could be responsible for genital herpes and HSV-2 could cause herpes labialis [6]. HSV is highly prevalent in humans. It is estimated that 70% and 10% of the global population is infected with HSV-1 and HSV-2, respectively. While the proportion of world population expected to have symptomatic HSV-1 and HSV-2 infections is approximately 16% and 5%, respectively [1]. Most commonly, the virus replication is limited to epithelial cells and establishes latency in enervating sensory neurons, where it can remain quiescent for an extensive period of time [2, 7–9]. Infection is lifelong, with reactivation of the latent virus causing subsequent outbreaks at the infection site. These are usually milder than the primary infection and lesions heal at 10 days if no treatment is undertaken [1, 3]. On average, reactivations occur six times a year [1], but frequency and severity decrease over time [2, 9]. The recurrence rate is higher for HSV-2 infection compared to HSV-1 [1, 5]. Several stimuli can trigger HSV reactivation, such as ultraviolet radiation, abnormal hormone levels, fatigue, stress and traumatic events, as well as immunosuppression [1, 10]. The development of herpes labialis and genital herpes lesions is sequential and begins with a prodrome showing erythema (usually accompanied by itching) [1, 2, 11], then papules, vesicles, ulcers or soft crust, hard crust, residual abnormalities (dry flaking skin and residual swelling and erythema), and finally reepithelization and healing (normal skin) [11]. Although HSV infections are usually benign [7], this virus can also cause severe diseases such as encephalitis, lymphocytic meningitis, blindness, and systemic disease in neonates and immunocompromised patients [7, 12]. While the diagnosis of HSV is often based on clinical presentation [5], laboratory confirmation is strongly recommended [2, 13].

Once infected with HSV, there is no cure. Treatment can, however, attenuate the clinical course, suppress recurrences, as well as reducing viral shedding and complications [2, 9]. In general, the prevention and treatment of HSV are managed with systemic or topical drugs [2]. At present, there are many antiviral drugs to treat HSV infections. The mainstay of antiviral therapy over the past 40 years has been nucleoside analogs such as acyclovir [1, 10, 14]. And while these drugs have provided substantial improvement in the treatment of HSV infections, the intensive use of nucleoside analogs has led to the emergence of drug-resistant strains, with their long-term toxicity posing a threat [7, 8, 15, 16]. Consequently, the development of new antiviral agents has gained much interest in recent years [10, 15, 17, 18]. Several natural products have shown antiviral effects against HSV, such as extracts, fractionated compounds, and isolated molecules obtained from plants, among others [1, 11, 15, 18]. Furthermore, many plant-derived extracts have been reported to inhibit HSV replication [1, 17]. Natural products with potential anti-HSV activity have the advantage of producing less resistance, side effects, and toxicity [10, 15].

Intensive research has been carried out in Georgia to develop new natural therapeutical products produced from endemic plants. Based on traditional medicine recipes, the latest biotechnological approaches have been applied to develop Lazolex® Gel (Iveriapharma, Tbilisi, Georgia) [19, 20]. Lazolex® Gel is an emollient gel for dermatological use composed of three different silicones and walnut extract. This study aimed to evaluate the efficacy and tolerance of Lazolex® Gel to treat mucocutaneous lesions caused by HSV.

## 2. Materials and Methods

### 2.1. Study Design

A single-center, single-arm, open-label, phase II clinical trial was conducted between November 2007 and December 2011 at the JSC Skin and Venereal Diseases Research Institute in Tbilisi, Georgia.

The study protocol was approved by the Ethical Issues Commission of the Georgian Ministry of Health. The research was conducted following the principles established in the Declaration of Helsinki and regulations issued by the State Pharmacological Committee of the Georgian Ministry of Health for phase II clinical trials.

### 2.2. Participants

From 27 November 2007, outpatients attending the Polyclinic Department of the JSC Skin and Venereal Diseases Research Institute, who had been diagnosed with herpes simplex mucocutaneous infections, were eligible to participate in the study. Recruitment was completed on 12 December 2008.

Participants were enrolled in the study based on the following criteria: (1) men or women aged 18–65, (2) diagnosis of acute or chronic herpes simplex mucocutaneous infection, (3) mild course of disease (defined as body temperature <37.2°C and without signs of general infirmity), (4) application of last treatment for herpes simplex infection >3 months, (5) available to cooperate during the study, and (6) provision of written informed consent. Exclusion criteria included the following: (1) abnormal laboratory results, (2) hypersensitivity to the product or its components, (3) pregnancy or breastfeeding, (4) acute/chronic renal or liver failure, (5) history of migraine, (6) organic brain lesion, (7) generalized anxiety disorder, (8) blood supply disturbance in the vertebrobasilar pool, (9) stage 3 essential hypertension, (10) concomitant acute or decompensated disease that could affect the study results, (11) intake of acyclovir, antibiotics, immunosuppressants, antimetabolites, or glucocorticosteroids during 3-month period prior to the study, and (12) concomitant participation in another clinical trial.

Criteria for withdrawal of patients from the clinical trial were the following: (1) individual intolerance, (2) severe adverse effects, (3) general worsening condition, (4) need for the prescription of other treatments during the treatment period of the clinical trial, and (5) patient refusal to continue participation.

The patients were divided into two groups according to the infectious agent: HSV-1 and HSV-2.

### 2.3. Intervention

Regardless of their diagnosis, all participants were assigned to receive topical treatment with Lazolex® Gel. Patients were given the gel at the end of the screening visit (day 0) by the attending physicians. The gel was applied to the lesion four times a day over a 10-day period, with the first application at 9 am and the fourth at 9 pm. Patients administered Lazolex® Gel to the affected areas themselves.

During the study, participants continued taking their usual medicines, and this information was duly recorded. Administration of the following medications was not allowed during the treatment period of the study: topical treatments, virucides, antimycotic agents, antibiotics, antimetabolites, glucocorticosteroids, and immunosuppressants.

### 2.4. Visit Schedule and Data Collection

The clinical trial included six visits: the screening visit (day 0), three visits during the treatment period (days 1, 5, and 10), and two visits during the follow-up period (day 20 and approximately four years after finishing the treatment).

The data evaluated by physicians during the trial are shown in Table 1. The symptoms monitored included itching, pain, and burning at the infection site, which were classified as 0 (absent), 1 (mild), 2 (moderate), and 3 (severe). Physical examination included body temperature, heart rate, blood pressure, cardiac and pulmonary auscultation, and examination of regional lymph nodes. The lesion is also reported in terms of size (affected area in cm^2^) and evidence of the following: erythema, edema, vesicles, erosion, and crusts. Laboratory tests included blood tests (blood count, biochemistry test including AST, ALT, bilirubin, and ammonia, as well as enzyme immunoassays for the detection of immunoglobulins M and G against HSV-1 and HSV-2) and urine tests. All data were recorded on individual patient registration forms.

The last visit consisted of a phone call to determine the frequency of outbreaks during the 4-year follow-up period and to find out the degree of satisfaction regarding the long-term effects of Lazolex® Gel. Patients received no additional Lazolex® Gel treatment during these 4 years.

Records and documentation related to the clinical trial were kept in the archives of the JSC Skin and Venereal Diseases Research Institute.

### 2.5. Evaluation of Efficacy and Tolerance Outcomes

Efficacy was assessed according to the following criteria: (1) course of the disease and (2) reduction in outbreak frequency. Lazolex® Gel was classified as effective if herpes lesions improved or healed after 10 days of treatment and as ineffective if the lesions did not improve after this period. The median effective time (ET50), defined as the time (in days) needed to reach complete recovery in 50% of the patients, was used as the main variable for Lazolex® Gel efficacy. Secondary efficacy outcomes included the degree of patient satisfaction with treatment efficacy (categorized as high, medium, or low).

Tolerance outcomes were evaluated using objective and subjective criteria. The objective criteria included the comparison of laboratory tests and physical examination before and after Lazolex® Gel treatment. The subjective criteria included complaints and symptoms reported by patients. In the event of adverse reactions, they were assessed by the attending physicians. Individual tolerance was categorized into very satisfactory (no clinically significant changes in physical examination or laboratory tests; no adverse reactions), satisfactory (insignificant changes in physical examination or laboratory tests, or mild adverse reactions that do not require a change in treatment), and unsatisfactory (significant changes in physical examination or laboratory tests and/or occurrence of adverse reactions that require the withdrawal of the product as well as prescribing treatment to address the adverse reaction).

### 2.6. Statistical Analysis

A sample size of 15 participants was established for each group according to Julious et al. recommendations for pilot studies [21].

Statistical analyses were performed using BioStat 2008 (AnalystSoft Inc., Alexandria, VA, USA). The analysis was per-protocol. ET50 was calculated both for subjective criteria (symptoms) and objective criteria (herpes lesions). In the case of the objective criteria, two ET50 were calculated separately regarding the stage of the lesions. Thus, an ET50 was estimated for erythema and edema clearance (considered as inflammatory signs), while another ET50 was calculated for vesicles and erosion disappearance. ET50 was obtained using linear regression analysis. Recurrence rates before and after treatment with Lazolex® were compared using Wilcoxon signed-rank test. Statistical significance was set at *p* < 0.05. For the assessment of laboratory results before and after treatment, means and standard deviations were calculated and descriptive statistics used.

## 3. Results

### 3.1. Patient Characteristics

A total of 30 subjects that were screened for eligibility met the inclusion criteria. According to the serological test results, 15 were infected with HSV-1 and 15 with HSV-2. All participants in the HSV-1 group were diagnosed with herpes labialis and all of the HSV-2 group had genital herpes. No patient withdrew from the study and all participants completed the follow-up.

The baseline characteristics of the 30 participants are shown in Table 2. One participant in the HSV-2 cohort was taking Tirozol® (Thiamazole) as their usual treatment for hyperthyroidism.

Among the participants with genital herpes, eight had lesions on the mucosa (seven on the glans and a woman on the labia minora) and seven had lesions on the skin (five on the prepuce, one on the labia majora, and one in the anal region).

### 3.2. Evaluation of Efficacy

As shown in Table 3, 14 patients (93%) with HSV-1 herpes labialis had itching and burning at the infection site before treatment. Among these, 10 (64%) reported that both itching and burning disappeared after 1 day of treatment. Similarly, 11 patients (73%) had pain before starting Lazolex® Gel treatment and, of these, 10 patients (91%) reported complete resolution of pain after 1 day of treatment. Nearly all patients experienced symptom relief a few minutes after applying the gel to the lesion. On day 5, 13 patients (87%) reported no symptoms at all. The ET50 to symptoms resolution was 1.97 days (95% confidence interval [CI]: 0.11–3.83). While all patients with HSV-2 genital herpes had itching, 14 (93%) also had burning, and 12 (80%) had pain at the infection site before treatment. On day 5, 14 patients (93%) reported no symptoms at all. The ET50 (95% CI) to symptoms resolution was 3.11 days (1.22–4.99).

**Figure 1:**
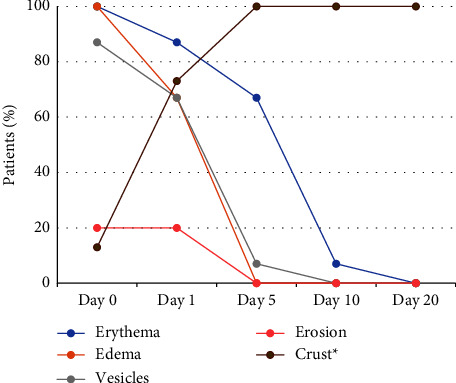
Evolution of the lesion stages during the treatment period of the study for the HSV-1 group (herpes labialis). The chart shows the proportion of patients with a corresponding type of lesion over a period of 20 days. *∗*Crust stage includes beginning of crust formation to the crust falling off.

**Figure 2:**
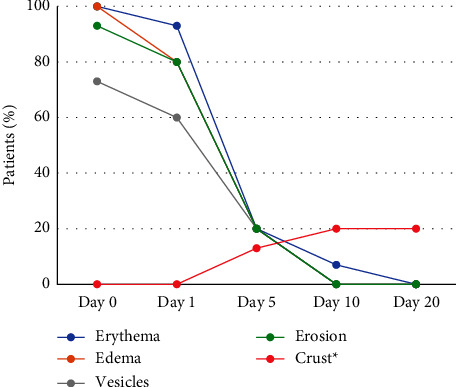
Evolution of the lesion stages during the treatment period of the study for the HSV-2 group (genital herpes). The chart shows the proportion of patients with a corresponding type of lesion over a period of 20 days. *∗*Crust stage includes beginning of crust formation to the crust falling off.

**Figure 3:**
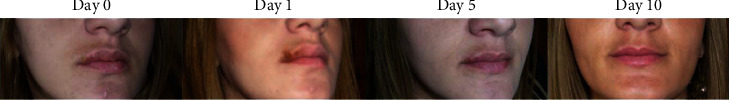
Visual chart for herpes labialis progression in a 27-year-old female infected with HSV-1.

**Figure 4:**
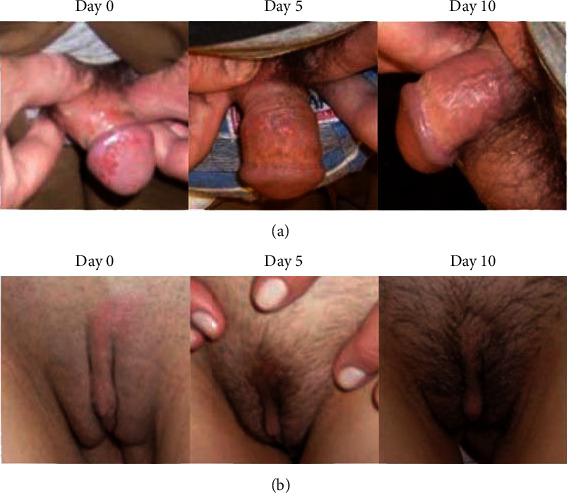
Visual chart for genital herpes progression. (a) 49-year-old male infected with HSV-2. (b) 46-year-old female infected with HSV-2.

Figure 1 shows the evolution of lesion stages during the study treatment period for the HSV-1 group (herpes labialis). On day 5, two patients out of 15 (13%) were at the beginning of the crust stage, in eight patients (53%), the crust was already formed, and in five (33%) patients, the crust had fallen off. Also on day 5, among the patients who had already formed the crust, one participant showed a new herpes simplex lesion on the chin. Complete healing was observed in 5 patients (33%) on day 5, and in 13 patients (87%) on day 10. The ET50 (95% CI) for vesicles and erosion to disappear was 3.64 days (2.45–4.83) and the ET50 (95% CI) for inflammation signs to disappear was 5.70 days (3.96–7.44). Similarly, Figure 2 shows the evolution of the stages of the lesion during the treatment period for the HSV-2 group (genital herpes). Complete healing was observed in 12 patients (80%) on day 5, and in all patients on day 10. The ET50 (95% CI) for vesicles and erosion to disappear was 3.88 (1.22–4.99) and the ET50 (95% CI) for inflammation signs to disappear was 4.32 (2.13–6.45). To illustrate these results, images showing the progression of the lesions during the treatment period are presented in Figures 3 and 4.

Regarding the rate of recurrence prior to the clinical trial, patients in the HSV-1 cohort (herpes labialis) had a median of 2 episodes per year (range 1–7). Interestingly, during the 4-year follow-up after treatment with Lazolex® Gel, the frequency of outbreaks was significantly reduced to 0.25 per year (range 0–1; *p*=0.001). Twelve patients (80%) classified the efficacy of Lazolex® Gel to reduce the number of HSV-1 outbreaks as high. Similarly, patients in the HSV-2 group (genital herpes) had a median of 1 episode per year (range 0–6). During the 4-year follow-up after treatment, the recurrence rate significantly decreased to 0.00 per year (range 0.00–0.25; *p*=0.003). Thirteen participants (87%) estimated the efficacy of Lazolex® Gel to reduce the number of HSV-2 outbreaks as high. Among the participants asked about the symptoms of outbreaks that occurred during the 4-year follow-up, five patients (63%) from the HSV-1 group (herpes labialis) and four (80%) from the HSV-2 group (genital herpes) reported that the intensity of symptoms was milder when compared to outbreaks prior to Lazolex® Gel treatment.

### 3.3. Assessment of Tolerance

Laboratory test results remained within the corresponding reference intervals both before (day 0) and after treatment (day 10). Prior to treatment, two patients had the enlargement of regional lymph nodes (one in the HSV-1 group and the other from the HSV-2 group), whereas after treatment their size was reported as normal.

In the HSV-1 group (herpes labialis) and the HSV-2 group (genital herpes), no side effects or adverse reactions were reported. Product tolerance was classified as highly satisfactory for the 15 patients in each group.

## 4. Discussion

The present study evaluates the efficacy and tolerance of Lazolex® Gel to treat herpes simplex mucocutaneous infections. Our findings show that Lazolex® Gel applied to lesions four times a day for 10 days is effective and safe in patients with HSV infections, as well as considerably reducing the rate of recurrence during a post-treatment period of 4 years.

As reported by patients, Lazolex® Gel treatment soothes symptoms associated with herpes labialis and genital herpes, such as itching, burning, and pain. Thus, according to our results, symptoms are expected to resolve in 50% of patients 2 and 3 days after treatment initiation for HSV-1 (herpes labialis) and HSV-2 (genital herpes), respectively. Furthermore, our results show that most patients will be free of symptoms after 5 days of treatment (in our study, 87% and 93% of patients with HSV-1 and HSV-2 mucocutaneous infections, respectively). It is worth mentioning that nearly all participants felt symptom relief a few minutes after applying the gel. The immediate effect of Lazolex® Gel could be greatly appreciated by patients since itching, burning, or pain are bothersome symptoms associated with HSV infections and their swift relief is highly welcomed [22].

In our study, the characteristics of lesions also started to improve on the very first day of treatment. Both vesicles and erosion disappeared in half of the patients on day 4 for both the HSV-1 (herpes labialis) and HSV-2 (genital herpes) groups. Similarly, the resolution of inflammatory signs (erythema and edema) took place on day 6 and day 4–5 in half of the patients infected with HSV-1 and HSV-2, respectively. By day 5, most patients treated with Lazolex® Gel are expected to achieve complete healing (in our study, 33% and 80% of patients with HVS-1 and HSV-2 infections, respectively).

Lazolex® Gel is an emollient gel for dermatological use that contains three different silicones and walnut extract. Silicones have been widely used in topical applications for decades [23]. They are considered safe and have countless benefits, such as being easy to spread, long-lasting, resistant to washing-off, biocompatible, producing an anti-inflammatory effect, and offering a film-forming ability that protects against chemical and microbial invasion [23–25]. Furthermore, silicones provide a pleasant, silky, nongreasy, nonstaining texture [25, 26]. All these benefits are due to the unique physicochemical properties of silicones: emollience, chemical and thermal stability, low glass transition temperature, low viscosity and near zero surface shear viscosity, low surface tension and moderate interfacial tensions, and high gas permeability [23, 25]. While many mechanisms of action have been proposed for silicones, it is universally accepted that they produce occlusion by forming a flexible hydrophobic barrier that decreases transepidermal water loss, and thus facilitates and maintains the hydration of the stratum corneum [23, 24, 27, 28]. Walnut extract, in contrast, has traditionally been used to treat ulcers, burns, and warts [29]. It has a polyphenol content that provides antioxidant, antimicrobial properties [30–32]. Lazolez® Gel acts by forming a film that protects the mucocutaneous lesions caused by HSV from dryness, irritation, and external agents. This barrier maintains a favorable environment for wound healing. By reducing small contaminants inside the lesion (dead cells, cellular debris, virus, and bacterial particles, among others), hydrating, and cleaning the wound, Lazolex® Gel reduces pain and irritation in addition to expediting healing.

Satisfactory results have been achieved with other topical products, both synthetic and natural, in the treatment of herpes simplex mucocutaneous infections. Boes et al. [33] compared three different products to treat herpes labialis in a randomized clinical trial: a film-forming patch, a set of semiocclusive hydrocolloid patches, and acyclovir 5% cream. The mean healing times (95% CI) in days were 9.30 (8.75–9.85), 9.67 (9.11–10.22), and 9.80 (9.30–10.30), respectively. In addition to walnut extract, used to manufacture Lazolex® Gel, several plant extracts have been shown to have antiviral effects. For example, extracts of *Aglaia odorata*, *Moringa oleifera*, and *Ventilago denticulate* have been shown to have antiviral activity against HSV in cell cultures and animal models [1]. Essential oils extracted from plants such as *Glechon spathulate*, *Glechon marifolia*, and those from the *Labiatae* and *Verbenaceae* families have been described to have antiviral activity against HSV in cell cultures as well [1]. However, clinical trials testing botanical extracts have yet to be carried out [34]. Lemon balm cream was evaluated in 66 individuals with recurrent herpes labialis in a randomized, double-blind, placebo-controlled study. Lesions healed faster with lemon balm cream compared to placebo, and patients had less pain and vesicles [34, 35]. The combination of sage and rhubarb extract was tested in a randomized placebo-controlled study that included 149 patients with herpes labialis. The patients were treated topically with acyclovir, sage extract, or a combination of sage and rhubarb extract. This combination was more effective than sage alone, and as effective as acyclovir. The mean healing times were 7.6 for sage cream, 6.7 days for rhubarb-sage cream, and 6.5 days for acyclovir cream [36]. A parallel controlled open-label randomized clinical study that included 756 adults compared kanuka honey (obtained from the kanuka tree) with acyclovir 5% for the treatment of herpes labialis. The efficacy of topical medical-grade kanuka honey was no different to topical acyclovir. The median (95% CI) healing times in days were 8 (8–9) for acyclovir and 9 (8–9) for kanuka honey [37]. However, to our knowledge, there are no published data regarding the long-term efficacy of natural products in treating herpes simplex mucocutaneous infections, so our study is groundbreaking in this regard.

While other topical antiviral agents such as acyclovir 5% cream (with or without hydrocortisone) and penciclovir 5% cream are not effective in preventing recurrent herpes labialis [38], our data showed that Lazolex® Gel dramatically reduced the number of infection outbreaks in the years following treatment for both herpes labialis and genital herpes. The frequency of outbreaks significantly decreased from a median of 2.00 and 1.00 times a year to 0.25 and 0.00 in the HSV-1 (herpes labialis) and HSV-2 (genital herpes) cohorts, respectively. The diagnosis of herpes has important lifelong psychological implications [2, 9] and the prevalence of reactivations is estimated at an average of six times per year [1], so these findings may have a very positive impact on patient quality of life. Furthermore, reducing the rate of recurrence could have favorable consequences for health systems. In this regard, Xia et al. [39] calculated the costs associated with HSV infection in emergency rooms in US hospitals. From 2006 to 2013, the costs were estimated at 543 million dollars, going from 45 million dollars in 2006 to 91 million in 2013, mainly due to a 24% increase in the number of patients.

In our study, most of the patients considered the treatment highly effective at the end of the 4-year follow-up period. Several patients who had outbreaks during these years stated that the intensity of symptoms was milder compared to those prior to receiving Lazolex® Gel treatment. However, it should be noted that herpes recurrences tend to be less frequent over time [9]. When assessing patient satisfaction, there are other factors to consider in addition to efficacy, such as aesthetic aspects. Gels are widely accepted as convenient therapeutic products. In this regard, as reported by Skulason et al., the majority of patients prefer transparent gels to white creams due to the aesthetic benefits they offer [40].

Although standard therapies with acyclovir and other synthetic drugs are available, the safety of these drugs is limited due to the development of adverse effects, such as renal failure, hepatitis, and anaphylaxis [8, 41]. Our findings confirmed that tolerance to treatment with Lazolex® Gel applied to lesions four times a day for 10 days was good. There were no adverse reactions or side effects in the HSV-1 group (herpes labialis) or HSV-2 group (genital herpes). Given these results and those of the toxicological studies previously performed by the manufacturer, Lazolex® Gel is considered safe. Novel treatment approaches for herpes simplex infections are needed not only because of the safety issues mentioned above, but also due to the emergence of virus strains resistant to commonly used antiviral drugs [8]. Thus, Lazolex® Gel could be an effective alternative treatment for herpes simplex mucocutaneous infections.

While this is the first study to investigate Lazolex® Gel as a treatment for herpes labialis and genital herpes, previous studies have evaluated its potential to treat recurrent stomatitis. In the comparative study by Gogotishvili et al. [19], which included 50 patients with recurrent aphthous stomatitis, Lazolex® Gel was more effective than treatment with Solcoseryl® dental adhesive paste, A and E vitamins, and cedar oil. Lazolex® Gel has been shown to accelerate tissue regeneration and increase the duration of remission in patients with recurrent aphthous stomatitis and recurrent herpetic stomatitis [20, 42]. Among these studies, the only one which focused on the safety profile for the treatment reported no adverse events, a finding consistent with the positive tolerance observed in our study [42].

The present study has several limitations. Firstly, the study was conducted with no control group. This pilot study was designed as an initial step to assess the efficacy and tolerance of Lazolex®. Thanks to the preliminary information this trial obtained, a randomized, double-blind, placebo-controlled study is currently underway to evaluate the effectiveness and safety of Lazolex® in patients with recurrent genital herpes (LAZOGENHER study). In the HSV-1 cohort (herpes labialis), the only sex represented was women (100%). This fact can be considered a limitation of the study, but can be explained by the higher prevalence of clinically manifested orofacial herpes simplex in females compared to males [33]. This clinical trial also offers important advantages, such as the evaluation of long-term efficacy. Furthermore, unlike other studies [33], the progression of the lesions was evaluated by an attending physician.

## 5. Conclusions

Topical treatment with Lazolex® Gel applied to lesions four times a day for 10 days has proven to be effective and safe to treat mucocutaneous lesions caused by HSV-1 and HSV-2 and to reduce the frequency of recurrence. It is effective in controlling pain and inflammation as well as shortening the duration of typical symptoms such as itching and burning. Lazolex® Gel is a promising candidate for the topical treatment of herpes infections, particularly given the emerging problem of drug resistance. It would be advisable to design more extensive clinical trials to confirm the ET50 reported in the present study. In addition, randomized placebo-controlled studies are needed, not only to confirm our findings, but also to reinforce the efficacy of plant extracts in treating HSV infections.

## Figures and Tables

**Table 1:** Visit schedule and assessed data during the trial.

Examination day	Screening period	Treatment period				Follow-up period
	Day 0	Day 1	Day 5	Day 10	Day 20	Subsequent 4 years
General information						
Age, sex	X					
Written informed consent signed	X					
Case history including frequency of outbreaks prior to study	X					
Objective assessment						
General physical examination	X	X	X	X	X	
Laboratory tests	X			X		
Assessment of tolerance				X	X	
Assessment of efficacy				X	X	X
Characteristics of HSV lesion						
Affected area (cm^2^)	X	X	X	X	X	
Lesion stage	X	X	X	X	X	
Occurring symptoms (itching, pain, burning)	X	X	X	X	X	
Photo documentation	X	X	X	X		
Subjective assessment						
Reporting of side effects		X	X	X	X	
Degree of satisfaction with treatment						X

HSV: Herpes simplex virus.

**Table 2:** Baseline characteristics of study participants.

Characteristic	Frequency (%)	
	HSV-1 herpes labialis group	HSV-2 genital herpes group
Female	15 (100)	3 (0)
Male	0 (0)	12 (80)
Median age (range)	32 (18–57)	38 (24–65)
Concomitant treatment for other conditions	0 (0)	1 (7)

HSV: Herpes simplex virus.

**Table 3:** Patients classified by symptoms intensity during the treatment phase.

	Day 0	Day 1	Day 5	Day 10	Day 20
HSV-1 herpes labialis group (*N* = 15)					
Itching					
Severe	2	0	0	0	0
Moderate	6	0	0	0	0
Mild	6	5	2	0	0
Absent	1	10	13	15	15
Burning					
Severe	2	0	0	0	0
Moderate	8	0	0	0	0
Mild	4	5	2	0	0
Absent	1	10	13	15	15
Pain					
Severe	0	0	0	0	0
Moderate	3	0	0	0	0
Mild	8	1	0	0	0
Absent	4	14	15	15	15

HSV-2 genital herpes group (*N* = 15)					
Itching					
Severe	1	2	0	0	0
Moderate	7	4	0	0	0
Mild	7	6	1	0	0
Absent	0	3	14	15	15
Burning					
Severe	1	1	0	0	0
Moderate	8	2	0	0	0
Mild	5	10	1	0	0
Absent	1	2	14	15	15
Pain					
Severe	1	1	0	0	0
Moderate	3	2	0	0	0
Mild	8	5	1	0	0
Absent	3	7	14	15	15

HSV: Herpes simplex virus.

## Data Availability

Data used to support the findings of this study are available from the corresponding author upon request.

## References

[1] Álvarez D. M., Castillo E., Duarte L. F. (2020). Current antivirals and novel botanical molecules interfering with herpes simplex virus infection. *Frontiers in Microbiology*.

[2] Cole S. (2020). Herpes simplex virus. *Nursing Clinics of North America*.

[3] James C., Harfouche M., Welton N. J. (2016). Herpes simplex virus: global infection prevalence and incidence estimates. *Bulletin of the World Health Organization*.

[4] Petti S., Lodi G. (2019). The controversial natural history of oral herpes simplex virus type 1 infection. *Oral Diseases*.

[5] Clebak K. T., Malone M. A. (2018). Skin infections. *Primary Care: Clinics in Office Practice*.

[6] Banerjee A., Kulkarni S., Mukherjee A. (2020). Herpes simplex virus: the hostile guest that takes over your home. *Frontiers in Microbiology*.

[7] Whitley R., Baines J. (2018). Clinical management of herpes simplex virus infections: past, present, and future. *F1000Research*.

[8] Schnitzler P. (2019). Essential oils for the treatment of herpes simplex virus infections. *Chemotherapy*.

[9] Crimi S., Fiorillo L., Bianchi A. (2019). Herpes virus, oral clinical signs and QoL: systematic review of recent data. *Viruses*.

[10] Li W., Wang X. H., Luo Z. (2018). Traditional Chinese medicine as a potential source for HSV-1 therapy by acting on virus or the susceptibility of host. *IJMS*.

[11] Rosa M. I. D., Souza S. L., Farias B. F. D., Pires P. D., Dondossola E. R., Reis M. E. F. D. (2015). Efficacy of topical 5% acyclovir-1% hydrocortisone cream (ME-609) for treatment of herpes labialis: a systematic review. *Anais da Academia Brasileira de Ciencias*.

[12] Annunziata G., Maisto M., Schisano C. (2018). Resveratrol as a novel anti-herpes simplex virus nutraceutical agent: an overview. *Viruses*.

[13] Gnann J. W., Whitley R. J. (2016). Genital herpes. *New England Journal of Medicine*.

[14] De S. K., Hart J. C. L., Breuer J. (2015). Herpes simplex virus and varicella zoster virus: recent advances in therapy. *Current Opinion in Infectious Diseases*.

[15] Hassan S. T. S., Masarčíková R., Berchová K. (2015). Bioactive natural products with anti-herpes simplex virus properties. *Journal of Pharmacy and Pharmacology*.

[16] Zhao M., Zheng R., Jiang J. (2015). Topical lipophilic epigallocatechin-3-gallate on herpes labialis: a phase II clinical trial of AverTeaX formula. *Oral Surgery, Oral Medicine, Oral Pathology and Oral Radiology*.

[17] Cagno V., Sgorbini B., Sanna C. (2017). In vitro anti-herpes simplex virus-2 activity of Salvia desoleana Atzei & V. Picci essential oil. *PLoS ONE*.

[18] Rechenchoski D. Z., Agostinho K. F., Faccin-Galhardi L. C. (2020). Mangiferin: a promising natural xanthone from *Mangifera indica* for the control of acyclovir—resistant herpes simplex virus 1 infection. *Bioorganic & Medicinal Chemistry*.

[19] Gogotishvili M., Abashidze N., Korsantia N., Korsantia N. (2021). Immunomodulatory and clinical effectivity of the drug «Lazolex» in treatment of recurrent aphthous stomatitis (RAS). *Journal of Experimental and Clinical Medicine Georgia*.

[20] Gogotishvili M., Abashidze N., Korsantia B. (2020). Study of antiviral and immunecorrective effects of Lazolex in patients with recurrent herpetic stomatitis. *Georgian Medical News*.

[21] Julious S. A. (2005). Sample size of 12 per group rule of thumb for a pilot study. *Pharmaceutical Statistics*.

[22] Herpes M.. (2008). Simplex virus: drug resistance and new treatment options using natural products (Review). http://www.spandidos-publications.com/mmr/article.jsp?article_id=mmr_1_5_611.

[23] Sounouvou H. T., Lechanteur A., Piel G., Evrard B. (2022). Silicones in dermatological topical drug formulation: overview and advances. *International Journal of Pharmaceutics*.

[24] Benedetto A. V. (2019). What’s new in cosmetic dermatology. *Dermatologic Clinics*.

[25] Aliyar H., Schalau G. (2015). Recent developments in silicones for topical and transdermal drug delivery. *Therapeutic Delivery*.

[26] Allen Loyd V. (2015). Basics of compounding: compounding with silicones. *IJPC*.

[27] Meaume S., Le Pillouer-Prost A., Richert B., Roseeuw D., Vadoud J. (2014). Management of scars: updated practical guidelines and use of silicones. *European Journal of Dermatology*.

[28] Angra K., Lipp M. B., Sekhon S., Wu D. C., Goldman M. P. (2021). Review of post-laser-resurfacing topical agents for improved healing and cosmesis. *Journal of Clinical and Aesthetic Dermatology*.

[29] Rodríguez-Archilla A., Raissouni T. (2017). Randomized clinical trial of the effectiveness of complementary therapies for recurrent aphthous stomatitis. *Medicina Clínica*.

[30] Croitoru A., Ficai D., Craciun L., Ficai A., Andronescu E. (2019). Evaluation and exploitation of bioactive compounds of walnut, juglans regia. *CPD*.

[31] Cheraghali F., Shojaee-aliabadi S., Hosseini S. (2018). Characterization of microcapsule containing walnut (*Juglans regia* L. green husk extract as preventive antioxidant and antimicrobial agent. *International Journal of Preventive Medicine*.

[32] Soto-Madrid D., Gutiérrez-Cutiño M., Pozo-Martínez J., Zúñiga-López M. C., Olea-Azar C., Matiacevich S. (2021). Dependence of the ripeness stage on the antioxidant and antimicrobial properties of walnut (juglans regia L.) green husk extracts from industrial by-products. *Molecules*.

[33] Boes H., Goulioumis V., Wechsler A., Zimmer S., Bizhang M. (2020). Clinical study on the effectiveness of three products in the treatment of herpes simplex labialis. *Scientific Reports*.

[34] Hoffmann J., Gendrisch F., Schempp C. M., Wölfle U. (2020). New herbal biomedicines for the topical treatment of dermatological disorders. *Biomedicines*.

[35] Koytchev R., Alken R. G., Dundarov S. (1999). Balm mint extract (Lo-701) for topical treatment of recurring herpes labialis. *Phytomedicine*.

[36] Saller R., Büechi S., Meyrat R., Schmidhauser C. (2001). Combined herbal preparation for topical treatment of herpes labialis. *Complement Med Res*.

[37] Semprini A., Singer J., Braithwaite I. (2019). Kanuka honey versus aciclovir for the topical treatment of herpes simplex labialis: a randomised controlled trial. *BMJ Open*.

[38] Leung A. K. C., Barankin B. (2017). Herpes labialis: an update. *Recent Patents on Inflammation & Allergy Drug Discovery*.

[39] Xia F., Fuhlbrigge M., Dommasch E., Joyce C., Mostaghimi A. (2018). Cost of routine herpes simplex virus infection visits to U.S. Emergency departments 2006-2013. *West*.

[40] Skulason S., Holbrook W. P., Thormar H., Gunnarsson G. B., Kristmundsdottir T. (2012). A study of the clinical activity of a gel combining monocaprin and doxycycline: a novel treatment for herpes labialis: clinical trial of a novel treatment for herpes labialis. *Journal of Oral Pathology & Medicine*.

[41] Gaby A. R. (2006). Natural remedies for herpes simplex. *Alternative Medicine Review*.

[42] Gogotishvili M. T., Abashidze N. O., Iverieli M. B., Gogishvili X. B., Gogebashvili N. G. (2014). The use of Lazolex during complex treatment of chronic recurrent aphthous stomatitis. *Tbilisi State Medical University Collection of Scientific Works*.

